# Solitary Cecal Diverticulitis: An Unusual Cause of Acute Right Iliac Fossa Pain—A Case Report and Review of the Literature

**DOI:** 10.1155/2014/131452

**Published:** 2014-11-23

**Authors:** Nikolaos Mudatsakis, Marinos Nikolaou, Konstantinos Krithinakis, Michail Matalliotakis, Nikolaos Politis, Emmanouil Andreadakis

**Affiliations:** ^1^Department of Surgery, General Hospital of Agios Nikolaos, Lasithi, 72100 Crete, Greece; ^2^Department of Gynecology, General Hospital of Agios Nikolaos, Lasithi, 72100 Crete, Greece; ^3^Department of Surgery, Hippokrateion General Hospital, Attiki, 11527 Athens, Greece

## Abstract

Solitary cecal diverticulitis is a rare cause of acute abdominal pain in the Western world. Its clinical presentation, in most cases, mimics acute appendicitis. A 38-year-old Caucasian man presented with acute abdomen and clinical signs of acute appendicitis. Laparotomy was performed and revealed an inflammatory, solitary diverticulum of the cecum. A typical appendectomy was performed and a catheter was inserted for draining percutaneously the inflamed diverticulum of the cecum. The patient had an uneventful recovery and was discharged on the 4th postoperative day. This frequently misdiagnosed condition, in most cases, is being suspected and identified intraoperatively as acute appendicitis. The aim of this study is to review the available different surgical management options and to present an alternative therapeutic approach that may be valuable under specific circumstances.

## 1. Introduction

The cecal diverticulum is a rare disease with reported incidence of 0.04% to 2.1% (1–3). The condition is uncommon in the Western countries, where 85% of diverticulum occurs more commonly in the descending and sigmoid colon rather than in cecum [1]. However, the cecal diverticulitis has a higher incidence (up to 71%) in Oriental populations [2]. The etiology of solitary cecal diverticulum remains still unclear, but its congenital origin seems possible as it includes all layers of the colonic wall. The average age for the development of this condition is about 43.6 years with male predominance [3]. The cecal diverticula are usually solitary and are situated in the area from 1 cm proximal to 2 cm distal to the ileocecal valve. Most of them arise from the anterior aspect of the cecum; therefore when inflamed they tend to perforate and cause acute, localized peritonitis. However, an acutely inflamed solitary cecal diverticulum is an uncommon cause of an acute abdomen [2, 4].

The disease is frequently misdiagnosed at the time of its occurrence. The symptoms and signs of the disease are well known to closely mimic acute appendicitis with abdominal pain, low-grade fever, nausea, vomiting, abdominal tenderness, and leukocytosis [5]. Most authors agree that only few of the patients are correctly diagnosed with acute diverticulitis preoperatively, since it is clinically indistinguishable from appendicitis and is often confused with carcinoma of the cecum during the operation [6].

Imaging studies on the other side could distinguish between right-sided diverticulitis and acute appendicitis with high accuracy and therefore they have the benefit of avoiding unexpected findings during the operation [7]. In most cases, the correct diagnosis of cecal diverticulitis is often made intraoperatively during exploration of suspected acute appendicitis [8]. However, even intraoperatively, the correct diagnosis is often indistinguishable from acute appendicitis due to extensive inflammation.

Controversies exist regarding the optimal management in nonperforated cecal diverticulitis, ranging from conservative approach with intravenous antibiotics to surgical procedures such as diverticulectomy and right hemicolectomy [9]. The management approach should be based on the clinical presentation of the patient, the intraoperative findings, and the surgeon's experience.

A rare case of a young man presenting with acute right, lower abdominal pain with solitary cecal diverticulum who was suspected preoperatively to have acute appendicitis is reported. The aim of the present case report was to review treatment strategies of this entity, since many issues still remain unclear, and to present an alternative therapeutic approach that may be valuable under specific circumstances.

## 2. Case Report

**Figure 1:**
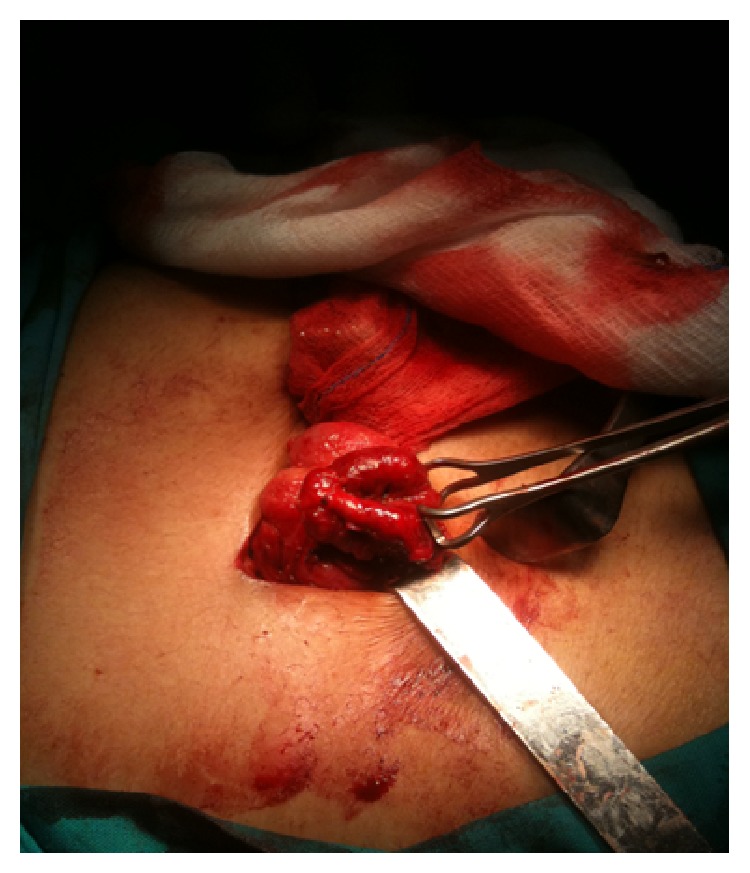
A solitary cecal diverticulitis.

A 38-year-old patient presented to the hospital's emergency department with a 24-hour history of a severe abdominal right, lower quadrant pain with accompanying anorexia, nausea, and vomiting. Physical examination revealed clinical signs of acute abdomen with local rebound tenderness. The vital signs were as follows: temperature 38.4°C, pulse rate 115 beats/min, blood pressure 120/60 mmHg, and respiratory rate 30/min. The laboratory test results on admission were within the normal limits except the elevated white blood cell (WBC) count of 19,000/*μ*L with 87 band forms. His past medical history was unremarkable. No preoperative imaging studies were performed because a presumptive diagnosis of acute appendicitis was made. The patient was transferred to the operative room for an emergency laparotomy and a standard open appendectomy through a McBurney incision was started. During the operation, a perforation of an acutely inflamed solitary diverticulum of cecum was found (Figure 1). The diameter of diverticulum was about 2 cm. Lateral to the cecum, an abscess was identified, walled off by the omentum and adjacent loops of ileum. The appendix was visualized and found to be normal. Since there was no suspicion of underlying malignancy, a conservative management was undertaken. After blunt mobilization of the omentum and the adherent loops of small bowel, the abscess was drained. The diverticulum was excised and, after trimming of its basis, we constructed a cecostomy with the insertion of a Petzer catheter into the cecum. An appendectomy was also performed as the last step of the operation. The postoperative course was otherwise uneventful. He recovered in the surgical ward on the following days and was discharged on the 4th postoperative day in good condition.

## 3. Discussion

Cecal diverticulitis is a rare clinical problem in the Western world, more commonly seen in the descending and sigmoid colon [2]. Right-sided colonic diverticulitis was first described in 1912 by Potier [10]. Subsequently, more than 500 cases of cecal diverticulitis have been reported in the literature. Solitary diverticulum of the cecum is believed to be congenital in origin and appears in the 6th week of pregnancy [8]. The majorities of them are developed in the frontal surface of the colon and are usually asymptomatic. In case of inflammation or perforation, the clinical symptoms and signs of the disease mimic acute appendicitis. Even during the operation sometimes the cecal diverticulitis is indistinguishable from acute appendicitis and carcinoma of the cecum [11].

The differential diagnosis of diverticulitis of the right colon is wide and includes acute appendicitis, gastroenteritis, urinary tract infection, ureteric calculi, pelvic inflammatory disease, cecum malignancy, foreign body perforation, ileocecal tuberculosis, and Crohn's disease [12]. Some studies suggest clinical features which could help in the differential diagnosis of cecal diverticulitis from acute appendicitis [3, 13]. In particular, there is greater duration of abdominal pain with lack of systemic toxic signs and low incidence of nausea and vomiting. The symptoms of cecal diverticulitis usually start and remain localized in the right iliac fossa rather than following the usual process of acute appendicitis originating in the epigastrium [14]. However, even if the above clinical symptoms strongly suggest diverticulitis of the right colon, it is frequently indistinguishable from acute appendicitis preoperatively and is often mistaken for carcinoma [15]. In a study of Chiu et al., the use of intraoperative colonoscopy for the differential diagnosis of cecal diverticulum and malignancy was suggested. During the time of appendectomy, a flexible endoscope was inserted via appendicular lumen and inspection of the region was made [16].

The preoperative diagnosis of disease is quite difficult without radiological imaging. Barium enema may be helpful in making the diagnosis, especially in patients with previous appendectomy and in those with more indolent symptoms [17]. Previous studies have reported that ultrasonography could differentiate between right-sided diverticulitis and acute appendicitis with high accuracy [18]. In particular, it was shown that ultrasonography had a sensitivity of 91.3% and a specificity of 99.8% with an overall accuracy of 99.5% in the diagnosis of cecal diverticulitis [4]. However, other researchers suggest that helical CT scan with intravenous contrast is superior to ultrasonography, as it can demonstrate features of acute right-sided diverticulitis with higher sensitivity [19]. Thus, the use of CT scanning for the evaluation of the right abdominal pain is very helpful for a careful preoperative assessment to exclude malignancy and to diminish the possibility of surgical intervention and patient's hospitalization [2, 5, 20, 21]. However, in approximately 10% of patients, diverticulitis is reported to be indistinguishable from carcinoma on a CT scan. Recently, magnetic resonance imaging (MRI) has been shown to be a useful tool in the diagnosis of right colonic diverticulitis, especially in young and pregnant patients [22].

The management of solitary diverticulitis of cecum is varied and controversial due to lack of randomized trials comparing conservative and aggressive surgical treatment methods (Table 1). If the diagnosis is established preoperatively, an expectant medical management is preferred. The conservative treatment approach with intravenous antibiotics and hydration can be applied in uncomplicated cecal diverticulitis [14, 21, 23]. If the diagnosis is established intraoperatively, for nonperforated diverticulitis of the right colon, appendicectomy combined with postoperative intravenous antibiotics is a safe and effective method for the treatment of cecal diverticulitis [9, 24]. However, the conservative treatment of diverticulitis has the risk of missing an inflammatory carcinoma on the right colon. Thus, this management option is more appropriate in Asian populations where benign pathology of the right colon cecal masses is much more common than neoplastic disease [13, 14].

There is no standard surgical procedure for the treatment of an acute, inflamed solitary cecal diverticulum, since the surgical approaches are not evidence-based. The choice of the surgical approach should be tailored to the operative findings and depends on the experience of the surgeon. Procedures range from simple isolated diverticulectomy and ileocecal resection to right hemicolectomy in patients with continued inflammation and suspicious malignancy [5].

Simple diverticulectomy with appendicectomy may be adequate if the intraoperative diagnosis of cecal diverticulitis is made with confidence. Diverticular resection can take place from appendicular incision; it has low rates of morbidity and mortality and is appropriate for solitary cecal diverticulitis. However, retrospective studies have demonstrated that resection of the cecal diverticulum is not appropriate for large inflammatory lesions and in cases suspected for malignancy. In such cases, there is a need for more aggressive surgical method like ileocecal resection and right hemicolectomy [14, 15, 31, 33]. In the case reported in this study, appendectomy and a cecostomy through a Petzer catheter were performed. In our point of view, this is an alternative therapeutic approach to the mentioned treatment options, especially in elderly and debilitated patients that may not tolerate such extensive procedures in the context of an underlying severe inflammatory process.

Other surgical treatment methods like the ileocecal resection have less surgical time than right hemicolectomy but cannot guarantee appropriate therapy in case of malignancy [4]. In the event of preoperative diagnostic uncertainty, a limited right hemicolectomy is the ideal therapy, especially in cases of extensive inflammation or when suspicious malignancy and granulomatous diseases cannot be ruled out [20, 27]. Particularly, in case of inflammatory mass, diverticular resection seems impossible and right hemicolectomy is suggested [15, 24, 31]. An emergency laparoscopic-assisted right hemicolectomy can be safely performed in patients with complicated cecal diverticulitis compared with the open approach, as it is associated with less blood loss and earlier return of bowel function [30]. After a literature review with 279 cases of surgical treatment of cecum diverticulum with ileocecal excision, no morbidity has been reported. On the contrary, the right hemicolectomy required increased surgical time and the morbidity percentage rate was high (up to 1.8%) [17, 24]. Subsequently, in patients with inflammatory cecal masses due to benign pathologies, this approach cannot be suggested. Fang et al. in a review of 85 patients with cecal diverticulitis recommend aggressive surgical resection as less than 40% of those patients who were treated conservatively had a successful outcome and no recurrence during follow-up period [24]. Similarly, another study by Lane et al. reported that 40% of patients treated with diverticular excision or intravenous antibiotic therapy required later hemicolectomy because of the continuous inflammatory process [5]. Additionally, a laparoscopic approach of the cecal diverticulitis has been reported as a safe and effective therapeutic option [25, 26, 28, 32]. However, the laparoscopic surgical treatment of uncomplicated cecal diverticulitis shows comparable results in the prevention of recurrence to conservative management with initial antibiotics [29]. Despite all the controversial management issues and the disagreement among surgeons, most of them suggest surgical excision of the inflamed cecal diverticulum with ileocecal resection [5, 8, 9, 14].

In conclusion, this case report illustrates that solitary diverticulitis of the cecum, even if it is a rare entity, should be taken into consideration during the differential diagnosis of patients complaining of right iliac fossa pain. The surgical trainees must be familiar with the diagnosis and management of this rare surgical disease. Preoperative imaging studies have been shown to correctly identifying this rare surgical disease. However, the surgeon should base his diagnosis on the operative findings as in most cases they mimic acute appendicitis.

## Figures and Tables

**Table 1:** Cases of cecal diverticulitis and therapeutic options.

Author	Number of patients	Treatment
Guven et al., 2014 [12]	12	Ileocecal resection/right hemicolectomy
Boselli et al., 2014 [20]	3	Right hemicolectomy
Kroening and Rai, 2013 [25]	1	Laparoscopic appendicectomy and excision of inflamed cecal diverticulum
Issa et al., 2012 [21]	15	Conservative medical therapy
Uwechue et al., 2012 [26]	1	Laparoscopic diverticulectomy
Paramythiotis et al., 2011 [27]	1	Right hemicolectomy
Altun et al., 2011 [28]	1	Laparoscopic right hemicolectomy
Kim et al., 2011 [23]	61	Conservative medical management
Park and Lee, 2010 [29]	132	Conservative therapy (102)/surgical management (30 patients)
Li et al., 2009 [30]	18	Right hemicolectomy
Griffiths and Date, 2007 [4]	1	Right hemicolectomy
Kurer, 2007 [2]	1	Right hemicolectomy
Ruiz-Tovar et al., 2006 [8]	5	Surgical management (ileocecal resection, right hemicolectomy)
Connonlly, 2006 [3]	3	Diverticulectomy/inversion of diverticulum
Abogunrin et al., 2005 [19]	1	Ileocecal resection and ileocolic anastomosis
Papapolychroniadis et al., 2004 [11]	11	Right hemicolectomy/conservative management
Fang et al., 2003 [24]	85	Conservative medical therapy (32 patients) and surgical management (67 patients)
Chedid et al., 2003 [31]	4	Conservative therapy (1 patient) and right hemicolectomy/ileotransverse anastomosis (3 patients)
Chiu et al., 2001 [9]	30	Appendicectomy and antibiotics
Lane et al., 1999 [5]	49	(Right hemicolectomy-80%, diverticulectomy-14%, appendectomy with drainage of intra-abdominal abscess-6%)
Pelosi III and Villalona, 1999 [32]	1 (pregnant)	Laparoscopic drainage, omental biopsy, and adhesiolysis
Harada and Whelan Jr., 1993 [13]	90	Surgical management (right colectomy/appendicectomy + antibiotics/diverticulectomy)
Fischer and Farkas, 1984 [15]	12	Appendicectomy and conservative management
